# Cost-effectiveness analysis of benmelstobart, anlotinib, and chemotherapy in extensive-stage small-cell lung cancer

**DOI:** 10.3389/fimmu.2024.1477146

**Published:** 2024-11-25

**Authors:** Maojin You, Lingling Luo, Tingting Lu, Shaofang Chen, Ying He

**Affiliations:** ^1^ Department of Pharmacy, Mindong Hospital Affiliated to Fujian Medical University, Ningde, China; ^2^ Department of Emergency Medicine, Mindong Hospital Affiliated to Fujian Medical University, Ningde, China

**Keywords:** cost-effectiveness, benmelstobart, anlotinib, chemotherapy, first-line treatment, extensive-stage small-cell lung cancer

## Abstract

**Background:**

The ETER701 trial assessed the efficacy and safety of benmelstobart combined with anlotinib plus etoposide/cisplatin (BEN-AL-EC) as a first-line therapy for extensive-stage small-cell lung cancer (ES-SCLC). Results indicated that BEN-AL-EC, when compared with placebo in combination with etoposide/cisplatin (PLB-EC), significantly enhanced both progression-free and overall survival rates, while demonstrating an acceptable safety profile among patients with ES-SCLC. However, BEN-AL-EC is expensive, necessitating its cost-effectiveness analysis.

**Methods:**

A Markov model with three health states was developed to evaluate the cost-effectiveness of BEN-AL-EC, AL-EC and PLB-EC for the treatment of ES-SCLC from the perspective of the Chinese healthcare system. Drug costs were derived from national tender prices, whereas other costs and utility values were derived from published literature. The key outcomes assessed included total costs, quality-adjusted life years (QALYs), and incremental cost-effectiveness ratios (ICERs). Sensitivity analyses, including one-way and probabilistic analyses, were performed to assess the robustness of the model.

**Results:**

The total cost of BEN-AL-EC was $55,117.42, yielding 1.09 QALYs, whereas that of PLB-EC was $15,238.15, yielding 0.71 QALYs. The ICER of BEN-AL-EC compared with PLB-EC was $106,249.42 per QALY gained. At a willingness-to-pay threshold of $38,133 per QALY, BEN-AL-EC had a 0% probability of being cost-effective relative to PLB-EC. The key parameters influencing these outcomes included utility values for PFS, the cost of benmelstobart, and the discount rate.

**Conclusion:**

From the perspective of the Chinese healthcare system, BEN-AL-EC as a first-line treatment for ES-SCLC is unlikely to be cost-effective when compared with PLB-EC.

## 1 Introduction

Small-cell lung cancer (SCLC) is a highly aggressive subtype of lung cancer, accounting for approximately 15% of all lung cancer cases (1). It is characterized by rapid cell proliferation and a tendency to quickly spread to other parts of the body, leading to a poor prognosis (2). More than 60% of patients with SCLC have extensive-stage disease at the initial diagnosis (3). Platinum-based chemotherapy combined with etoposide is the standard first-line treatment for extensive-stage SCLC (ES-SCLC); however, most patients have a median survival of only 9–11 months after treatment (4). Moreover, recent clinical studies have shown that the addition of immune checkpoint inhibitors (ICIs) to platinum-based chemotherapy increases the median survival of patients with ES-SCLC by only 2–4 months (5–8). Therefore, developing novel treatment strategies for ES-SCLC is necessary.

Anti-angiogenic drugs, such as anlotinib, can synergize with ICIs, thereby enhancing the therapeutic efficacy of the latter in various cancers (9, 10). Recently, a phase III clinical trial (ETER701) evaluated the efficacy and safety of benmelstobart, an ICI, combined with anlotinib plus etoposide/cisplatin (BEN-AL-EC) as a first-line treatment for ES-SCLC. The results indicated that compared with placebo combined with etoposide/cisplatin (PLB-EC), BEN-AL-EC significantly increased the median progression-free survival (PFS) (4.2 months versus 6.9 months) and overall survival (OS) (11.9 months versus 19.3 months) in patients with ES-SCLC, with an acceptable safety profile (11).

Although BEN-AL-EC may prolong the survival of patients with ES-SCLC, its cost-effectiveness should be carefully considered when adopting it as a therapeutic regimen, as it is inevitably more expensive than PLB-EC. To date, the cost-effectiveness of BEN-AL-EC as a first-line treatment for ES-SCLC has not been evaluated. Therefore, this study aims to assess the cost-effectiveness of BEN-AL-EC, relative to PLB-EC, as a first-line therapeutic strategy for ES-SCLC in the context of the Chinese healthcare system. The findings are reported by the CHEERS 2022 guidelines (**Supplementary Table A**) (12).

## 2 Methods

### 2.1 Model construction

**Figure 1:**
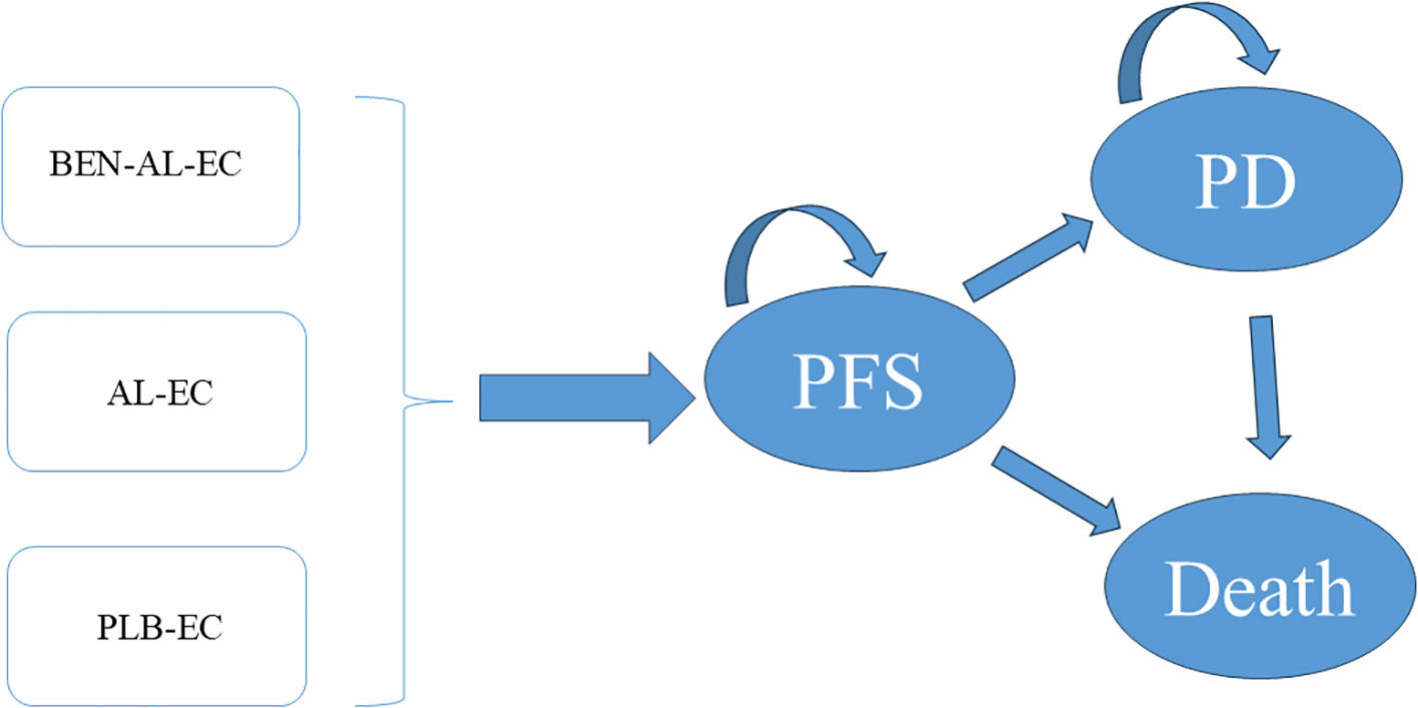
The Markov model simulating outcomes for the ETER701 trial. All patients started with PFS state and received treatment with BEN-AL-EC, AL-EC, or PLB-EC. AL-EC, anlotinib plus etoposide/carboplatin; BEN-AL-EC, benmelstobart combined with anlotinib plus etoposide/carboplatin; PD, disease progression; PFS, progression-free survival; PLB-EC, placebo combined with etoposide/cisplatin.

The TreeAge Pro 2022 software was used to construct a Markov model to assess the cost-effectiveness of BEN-AL-EC, anlotinib plus etoposide/cisplatin (AL-EC), and PLB-EC in the treatment of ES-SCLC (**Figure 1**). The model incorporated three health states, namely, PFS, disease progression (PD), and death. The length of each cycle of the model was set at 21 days, totaling 130 cycles over an approximate duration of 7.5 years, by which 99% of patients were expected to be deceased. We assume that all patients enter the model in a PFS state (13). During the model’s operation, patients exhibit a unidirectional health trajectory, where they can either sustain their present health status or transition to a subsequent state of health improvement, without the possibility of regression to a prior health condition. We incorporate background mortality rates from China into the model as well (14). Model outcomes included the total costs, quality-adjusted life years (QALYs), and incremental cost-effectiveness ratios (ICERs). Consistent with the Chinese Pharmacoeconomic Evaluation Guideline (15), the willingness-to-pay (WTP) threshold was set at three times the 2023 Chinese per capita GDP (i.e., $38,133 per QALY). A treatment strategy was considered cost-effective if its ICER was below this threshold.

### 2.2 Clinical information

Data on drug efficacy and safety were obtained from the ETER701 trial (11), a randomized, double-blind, phase 3 clinical trial conducted at 72 centers across China. The inclusion criteria were as follows: 1) patients aged 18–75 years and 2) patients who had histologically confirmed ES-SCLC and did not previously receive systemic therapy. Eligible patients were randomly allocated to the BEN-AL-EC, AL-EC or PLB-EC treatment regimen. Patients preferred to undergo 4 cycles of induction therapy, with each cycle lasting 3 weeks. Patients who achieved a complete or partial response and those who had stable disease with manageable toxicity subsequently received maintenance therapy. During induction therapy, benmelstobart was administered intravenously at a dose of 1200 mg on day 1 of each cycle, anlotinib orally at 12 mg once daily for the first 2 weeks of each cycle, etoposide intravenously at 100 mg/m^2^ on days 1–3 of each cycle, and carboplatin intravenously at an area under the curve of 5 mg/mL/min on day 1 of each cycle. During maintenance therapy, patients in the BEN-AL-EC group continued to receive benmelstobart and anlotinib, and patients in the AL-EC group continued to receive anlotinib, whereas those in the PLB-EC group received the placebo until PD or unacceptable toxicity occurred. Following PD or unacceptable toxicity in patients, we assume that some patients undergo chemotherapy as second-line treatment, while others receive the best supportive care (BSC). According to the ETER701 trial (11), in the BEN-AL-EC group, the median time to discontinuation of benmelstobart due to drug toxicity was 1.45 months, and for anlotinib it was 1.38 months. In the AL-EC group, the median time to discontinuation of anlotinib due to drug toxicity was 1.25 months. In the PLB-EC group, the median time to discontinuation of placebo due to drug toxicity was 1.35 months.

### 2.3 Survival or transition probabilities

The GetData Graph Digitizer (version 2.26) was used to extract PFS and OS data from the Kaplan–Meier survival curves reported in the ETER701 trial and reconstruct individual patient data. As described by Hoyle et al. (16), various survival distributions were fitted to the reconstructed individual patient data using the R software to generate survival curves beyond the follow-up period reported in the trial. These distributions included exponential, gamma, gen.F, gen. gamma, Gompertz, Weibull, log-logistic, and log-normal (**Supplementary Table B**). Based on Akaike and Bayesian information criteria (17, 18), we selected the log-logistic distribution as the best fit for the original survival curves (**Supplementary Figure A**, **Table 1**), which enabled the estimation of transition probabilities between different health states.

**Table 1:** Economic parameters of the model and the range of sensitivity analysis.

Variable	Base Value	Range		Distribution	Reference
		Min	Max		
Log-logistic distribution of PFS					
BEN-AL-EC	Scale = 0.1279405, Shape = 2.386172	–	–	–	Model fitting
AL-EC	Scale = 0.1550655, Shape = 2.967729	–	–	–	Model fitting
PLB-EC	Scale = 0.2143154, Shape = 3.964032	–	–	–	Model fitting
Log-logistic distribution of OS					
BEN-AL-EC	Scale = 0.05507908, Shape = 1.816331	–	–		Model fitting
AL-EC	Scale = 0.07095725, Shape = 2.144477	–	–		Model fitting
PLB-EC	Scale = 0.07892053, Shape = 2.269792	–	–		Model fitting
BEN-AL-EC group: Incidence of AEs (%)					
Neutropenia	69.5	55.6	83.4	Beta	(11)
Leukopenia	38.2	30.6	45.8	Beta	(11)
Thrombocytopenia	49.6	39.7	59.5	Beta	(11)
Anemia	24.0	19.2	28.8	Beta	(11)
Hypertension	15.5	12.4	18.6	Beta	(11)
AL-EC group: Incidence of AEs (%)					
Neutropenia	73.0	58.4	87.6	Beta	(11)
Leukopenia	30.7	24.6	36.8	Beta	(11)
Thrombocytopenia	53.7	43.0	64.4	Beta	(11)
Anemia	26.6	21.3	31.9	Beta	(11)
Hypertension	11.9	9.5	14.3	Beta	(11)
PLB-EC group: Incidence of AEs (%)					
Neutropenia	68.7	55.0	82.4	Beta	(11)
Leukopenia	34.6	27.7	41.5	Beta	(11)
Thrombocytopenia	35.8	28.6	43.0	Beta	(11)
Anemia	23.6	18.9	28.3	Beta	(11)
Hypertension	1.6	1.3	1.9	Beta	(11)
Cost ($)					
Benmelstobart (1200 mg)	1746.8	1397.4	2096.2	Gamma	(19)
Anlotinib (12 mg)	40.3	32.2	48.4	Gamma	(19)
Etoposide (100 mg)	1.1	0.9	1.3	Gamma	(19)
Carboplatin (100 mg)	7.3	5.8	8.8	Gamma	(19)
Neutropenia	83.5	66.8	100.2	Gamma	(20)
Leukopenia	211.3	169.0	253.5	Gamma	(21)
Thrombocytopenia	1081.5	865.2	1297.8	Gamma	(22)
Anemia	104.6	83.7	125.5	Gamma	(23)
Hypertension	1.5	1.2	1.8	Gamma	(24)
BSC per cycle	182.6	146.1	219.1	Gamma	(25)
Routine follow-up per cycle	73.9	59.1	88.7	Gamma	(25)
Tests per cycle	358.1	286.5	429.7	Gamma	(26)
End-of-life care	1492.5	1194.0	1791.0	Gamma	(26)
Utility value					
PFS	0.673	0.5384	0.8076	Beta	(20)
PD	0.473	0.3784	0.5676	Beta	(20)
Utility decrement					
Neutropenia	-0.20	-0.16	-0.24	Beta	(27)
Leukopenia	-0.20	-0.16	-0.24	Beta	(27)
Thrombocytopenia	-0.19	-0.15	-0.23	Beta	(27)
Anemia	-0.073	-0.058	-0.088	Beta	(27)
Hypertension	-0.040	-0.032	-0.048	Beta	(27)
Creatinine clearance rate (mL/min)	70	56	84	Normal	(28)
Body surface area (m^2^)	1.72	1.38	2.06	Normal	(25)
Discount rate	0.05	0.00	0.08	Fixed	(15)
Proportion					
Receiving chemotherapy in the BEN-AL-EC group	0.329	0.263	0.395	Beta	(11)
Receiving chemotherapy in the AL-EC group	0.465	0.372	0.558	Beta	(11)
Receiving chemotherapy in the PLB-EC group	0.571	0.457	0.685	Beta	(11)

AE, adverse event; AL-EC, anlotinib plus etoposide/carboplatin; AL-EC, anlotinib plus etoposide/carboplatin; BEN-AL-EC, benmelstobart combined with anlotinib plus etoposide/carboplatin; BSC, the best supportive care; PD, disease progression; PLB-EC, placebo combined with etoposide/cisplatin; PFS, progression-free survival.

### 2.4 Costs and utilities

This study focused exclusively on direct medical expenses, encompassing the costs of medications, tests, routine follow-up, BSC, management of grade ≥3 adverse events occurring at rates exceeding 5%, and end-of-life care (**Table 1**). The costs of drugs were determined based on national tender prices, whereas data on other expenses were obtained from published literature and adjusted to reflect the values in 2023 using the medical price index of the National Bureau of Statistics of China (14). All costs were calculated in US dollars and converted to CNY (1 USD = 7.03 CNY, for the year 2023). Given the lack of quality-of-life data from the ETER701 trial, utility values for PFS and PD were obtained from existing Chinese studies (20). Additionally, we accounted for disutilities associated with grade ≥3 adverse events occurring at rates exceeding 5% to mitigate the potential bias resulting from the inclusion of identical utility values for both treatment groups in the model. All costs and utilities were discounted at 5% (29).

### 2.5 Sensitivity analysis of BEN-AL-EC compared with PLB-EC

One-way and probabilistic sensitivity analyses were used to validate the robustness of the model. In one-way sensitivity analysis, parameter values were adjusted within their reported 95% confidence intervals. For parameters lacking specific data, benchmark values were varied by ±20%. The discount rate ranged from 0% to 8% (**Table 1**). The results were visualized on tornado plots. To evaluate the influence of parameter uncertainty on the outcomes, a total of 1,000 Monte Carlo simulations were performed based on predefined distributions (**Table 1**). Scatter plots were generated to visualize the results of probabilistic sensitivity analysis. Furthermore, we recalculated the ICER comparing BEN-AL-EC with PLB-EC while systematically reducing the price of benmelstobart.

### 2.6 Scenario analysis of BEN-AL-EC compared with PLB-EC

Scenario 1 involved varying the duration of the model to 2, 4, and 6 years to evaluate its impact on the results. In Scenario 2, we assumed that only 30% or 50% of patients received subsequent therapies after PD to simulate the real-life phenomenon that some patients discontinue treatment for various reasons in clinical settings.

## 3 Results

### 3.1 Basic analysis results

The BEN-AL-EC group achieved 1.09 QALYs at a cost of $55,117.42, the AL-EC group achieved 0.83 QALYs at a cost of $22,936.64, and the PLB-EC group achieved 0.71 QALYs at a cost of $15,238.15. The incremental efficacy of BEN-AL-EC compared with PLB-EC was 0.38 QALYs, with an incremental cost of $39,879.28. Similarly, AL-EC had an incremental efficacy of 0.12 QALYs and an incremental cost of $7,698.49 compared with PLB-EC. The ICER per QALY was estimated to be $106,249.42 for BEN-AL-EC compared with PLB-EC and $66,733.19 per QALY for AL-EC compared with PLB-EC (**Table 2**). At the WTP threshold of $38,133 per QALY, neither BEN-AL-EC nor AL-EC was considered cost-effective as a first-line therapeutic strategy for the treatment of ES-SCLC in China compared with PLB-EC.

**Table 2:** Results of the cost-effectiveness analysis.

Regimen	Total cost ($)	Total QALYs	Incremental cost ($)	Incremental QALYs	ICER ($/QALY)
PLB-EC	15,238.15	0.71	–	–	–
AL-EC	22,936.64	0.83	7,698.49	0.12	66,733.19
BEN-AL-EC	55,117.42	1.09	39,879.28	0.38	106,249.42

AL-EC, anlotinib plus etoposide/carboplatin; BEN-AL-EC, benmelstobart combined with anlotinib plus etoposide/carboplatin; ICER, incremental cost-effectiveness ratio; PLB-EC, placebo combined with etoposide/cisplatin; QALY, quality-adjusted life year.

### 3.2 Sensitivity analysis of BEN-AL-EC compared with PLB-EC

**Figure 2:**
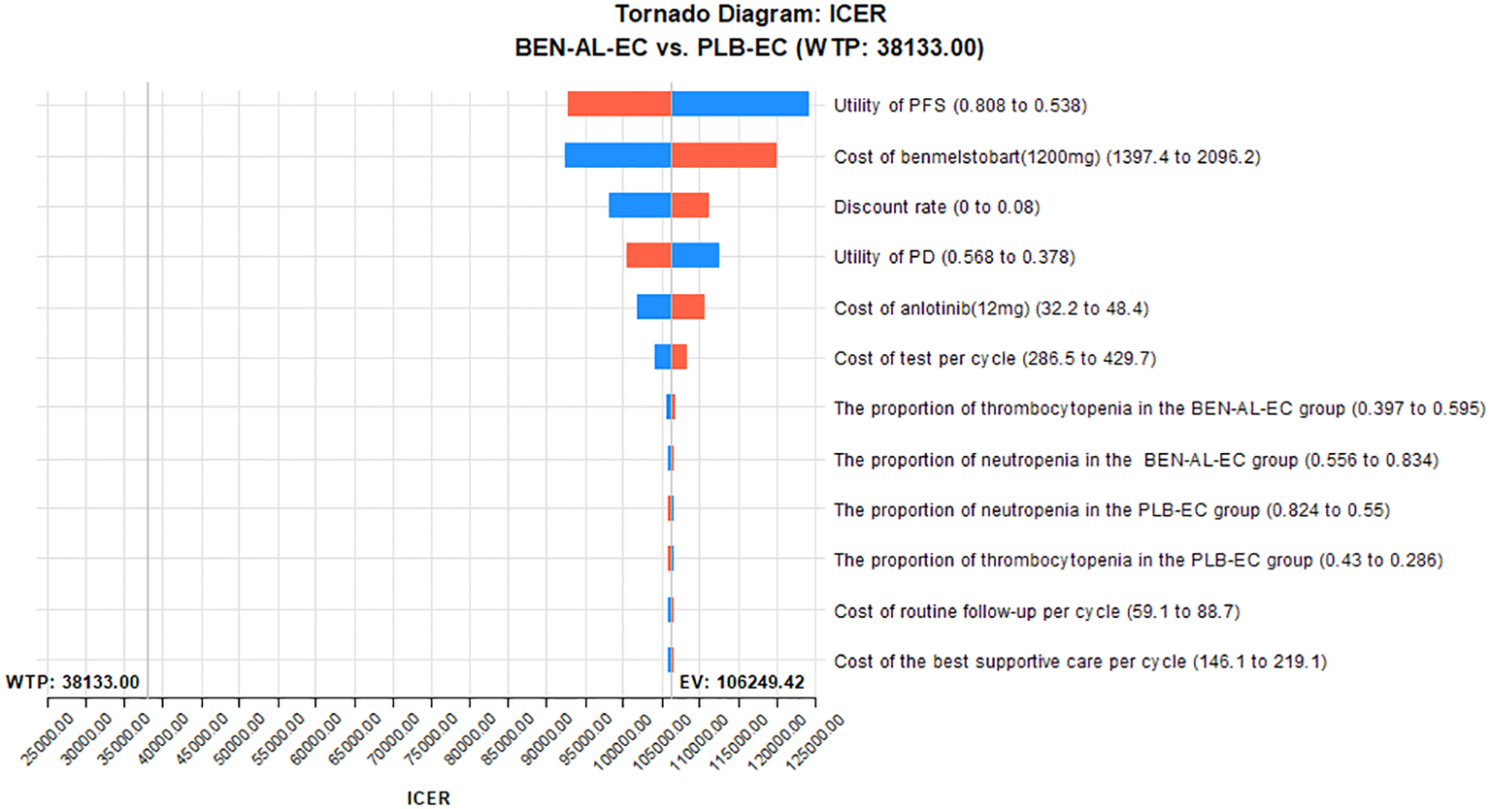
One-way sensitivity analyses of BEN-AL-EC in comparison with PLB-EC. BEN-AL-EC, benmelstobart combined with anlotinib plus etoposide/carboplatin; ICER, incremental cost-effectiveness ratio; PD, disease progression; PFS, progression-free survival; PLB-EC, placebo combined with etoposide/cisplatin; WTP, willingness-to-pay.

**Figure 3:**
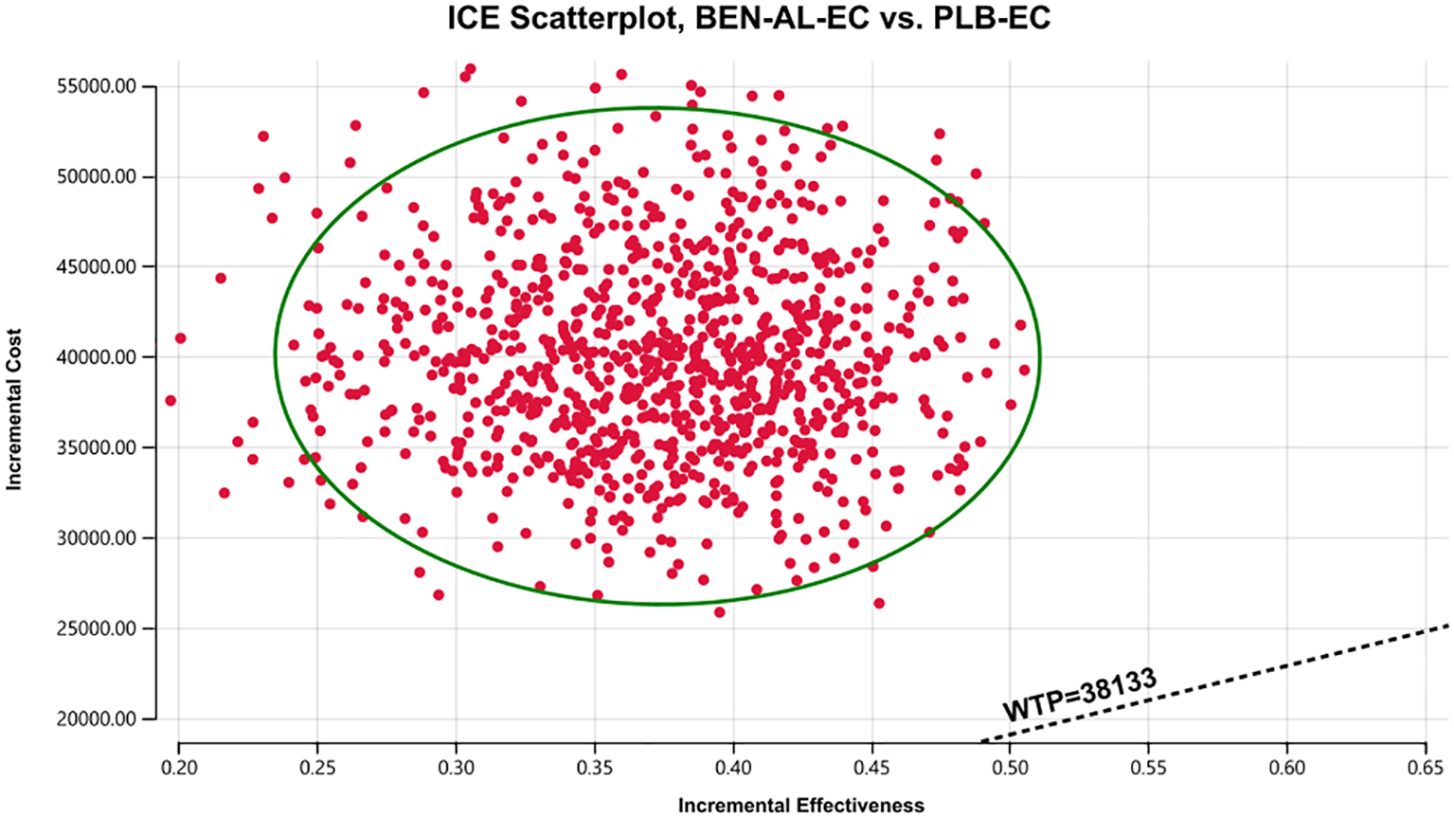
A probabilistic scatter plot of the ICER between the BEN-AL-EC and PLB-EC. Each point means the ICER for 1 simulation. Ellipses are used to indicate 95% confidence intervals. Points that lie below the ICER threshold represent cost-effective simulations. BEN-AL-EC, benmelstobart combined with anlotinib plus etoposide/carboplatin; ICE, incremental cost-effectiveness; PLB-EC, placebo combined with etoposide/cisplatin; WTP, willingness-to-pay.

The results of the one-way sensitivity analysis are presented in the tornado plot (**Figure 2**). In particular, the parameters that had the greatest impact on the outcomes of the model are the utility values of PFS, cost of benmelstobart, and discount rate. However, even if these parameters are varied within a certain range, the ICER is always higher than the predefined WTP threshold. This finding indicated that changes in the aforementioned parameters did not alter the outcomes of the model, whereas the influence of other parameters on the outcomes was relatively minor. The results of probabilistic sensitivity analysis are shown in the scatter plot in **Figure 3**. At a WTP threshold of $38,133, BEN-AL-EC had a 0% probability of being cost-effective when compared with PLB-EC. BEN-AL-EC may become a cost-effective treatment strategy if the price of benmelstobart (1200 mg) decreases below $24.1.

### 3.3 Scenario analysis of BEN-AL-EC compared with PLB-EC

The results of the scenario analysis are shown in **Table 3**. In scenario 1, when the duration of the model was changed to 2, 4, and 6 years, the ICER of BEN-AL-EC compared with PLB-EC was $195,270.13/QALY, $127,592.74/QALY, and $111,530.15/QALY, respectively. As the duration increased, the ICER gradually decreased. In scenario 2, when the proportion of patients receiving subsequent therapies was 30% and 50%, the ICERs were $101,794.35/QALY and $103,067.23/QALY, respectively, showing a minimal difference.

**Table 3:** Results of scenario analysis.

Scenarios	Cost ($)		QALY		ICER ($/QALY)
	BEN-AL-EC	PLB-EC	BEN-AL-EC	PLB-EC	
Scenario 1					
Model runtime (year) = 2	45,868.92	12,824.88	0.77	0.61	195,270.13
Model runtime (year) = 4	52,105.46	14,588.93	0.98	0.68	127,592.74
Model runtime (year) = 6	54,298.16	15,078.44	1.06	0.71	111,530.15
Scenario 2					
30% of total patients	46,703.07	8,495.94	1.09	0.71	101,794.35
50% of total patients	49,107.17	10,422.29	1.09	0.71	103,067.23

BEN-AL-EC, benmelstobart combined with anlotinib plus etoposide/carboplatin; ICER, incremental cost-effectiveness ratio; PLB-EC, placebo combined with etoposide/cisplatin; QALY, quality-adjusted life year.

## 4 Discussion

The ETER701 trial showed that compared with PLB-EC, BEN-AL-EC remarkably prolonged PFS and OS in patients with ES-SCLC. These clinical benefits of BEN-AL-EC, coupled with its manageable safety profile, highlight its potential as a new first-line treatment strategy for ES-SCLC. However, the high cost of BEN-AL-EC may substantially limit its widespread application, particularly among economically disadvantaged patients. Therefore, the primary objective of this study was to assess the cost-effectiveness of BEN-AL-EC as a first-line treatment strategy for ES-SCLC in the context of the Chinese healthcare system. The results showed that compared with PLB-EC, BEN-AL-EC incurred an additional cost of $106,249.42 per QALY, which largely exceeded the predefined WTP threshold of $38,133 per QALY. Therefore, BEN-AL-EC as a first-line treatment for ES-SCLC is not cost-effective in China. The lack of cost-effectiveness of the BEN-AL-EC regimen in treating ES-SCLC stems from its requirement for long-term maintenance therapy with benmelstobart and anlotinib, both of which are considerably more expensive than etoposide/cisplatin. This significantly increases the overall treatment costs of BEN-AL-EC without yielding sufficient incremental survival benefits. However, the cost-ineffectiveness of the BEN-AL-EC scheme should not be a reason to restrict its use, as this could deny patients the opportunity to benefit from a proven effective treatment. We should build upon the analysis results and seek multifaceted solutions. This includes exploring methods to reduce treatment costs, such as negotiating prices or seeking support from medical insurance to alleviate the financial burden on patients. Since the establishment of the National Health Security Bureau of China in 2018, multiple rounds of drug price negotiations with pharmaceutical companies have been conducted through the national procurement strategy to alleviate the medical burden on patients with cancer. Consequently, the prices of many anticancer drugs have decreased by 30–70% (30). Similarly, the price of benmelstobart, approved for marketing in China in May 2024, may be reduced through negotiation. On adjusting the price of benmelstobart to assess its impact on cost-effectiveness, we found that BEN-AL-EC might be used as a cost-effective first-line treatment strategy for ES-SCLC if the price of benmelstobart (1200 mg) decreases below $24.1. The findings of this study provide an important economic reference for negotiating the price of benmelstobart in China.

It should be noted that the results of this study indicate that AL-EC, as a first-line regimen for the treatment of ES-SCLC in China, is more cost-effective compared with PLB-EC than BEN-AL-EC, but it still higher our preset WTP value. Moreover, the survival benefit of AL-EC for treating ES-SCLC is not significant compared with PLB-EC (11). Therefore, AL-EC is not recommended as a first-line treatment for ES-SCLC, considering both its efficacy and cost-effectiveness.

The results of the one-way sensitivity analysis showed that parameters such as the utility value of PFS, cost of benmelstobart, and discount rate had a significant impact on the outcomes of the model. Notably, varying these parameters within their specified ranges did not alter the outcomes. Probabilistic sensitivity analysis indicated that BEN-AL-EC as a first-line treatment for ES-SCLC had a 0% probability of being cost-effective when compared with PLB-EC. These results indicate that our modeling results are robust. In addition, we conducted scenario analysis to cover two real-life clinical practice scenarios, thereby enhancing the applicability and generalizability of the findings. In scenario 1, we found more than 80% of the cost of the BEN-AL-EC regimen was incurred during the first 2 years of treatment and the ICER values gradually decreased as the treatment duration increased. Therefore, once patients are initiated on BEN-AL-EC, they should try to adhere to the regimen, as the cost-effectiveness gradually increases with the continuation of treatment. In scenario 2, the ICER values for BEN-AL-EC compared with PLB-EC remained stable as the number of patients receiving subsequent therapies increased following disease progression. This suggests that opting for subsequent treatments after disease progression does not diminish the cost-effectiveness of BEN-AL-EC. This finding encourages patients to continue treatment after disease progression rather than discontinue it. Such results are likely to be well-received by both physicians and patients because they meet ethical and moral requirements.

This study should emphasize several strengths. Firstly, to our knowledge, it is the first evaluation from the perspective of the Chinese healthcare system assessing the cost-effectiveness of BEN-AL-EC as a first-line regimen for treating ES-SCLC compared with PLB-EC. This will provide a significant reference value for China and other countries. This represents the most important strengths and innovative aspects of this study. Second, although BEN-AL-EC was not found to be cost-effective when compared with PLB-EC, it notably demonstrated an improvement in QALYs in patients with ES-SCLC (1.09 versus 0.71 QALYs). Third, the inclusion of only Chinese patient cohorts in the ETER701 trial enhanced the applicability of the findings of this study specifically to Chinese populations. However, this study has several limitations that should be acknowledged. First, due to the fact that our study is based on the results of the ETER 701 trial, we did not include other ICIs that have been demonstrated to be clinically effective for ES-SCLC, such as atezolizumab. This may limit the comprehensiveness of our findings, as chemotherapy combined with ICIs is currently regarded as the standard treatment for ES-SCLC. Second, our reliance on survival models to simulate data beyond the follow-up period of the trial might have introduced inherent bias compared with actual long-term survival data. We intend to update this cost-effectiveness analysis as long-term survival data becomes available. Third, the model developed in this study accounted for only grade ≥3 adverse events with an incidence exceeding 5%. However, sensitivity analysis indicated that variations in the probabilities of adverse events did not significantly affect the outcomes of the model.

## 5 Conclusion

From the perspective of the Chinese healthcare system, BEN-AL-EC as a first-line treatment strategy is not cost-effective when compared with PLB-EC for the treatment of ES-SCLC. A substantial reduction in the price of benmelstobart is required to make BEN-AL-EC cost-effective.

## Data Availability

The original contributions presented in the study are included in the article/**Supplementary Material**. Further inquiries can be directed to the corresponding author.

## References

[1] Rudin CM Brambilla E Faivre-Finn C Sage J. Small-cell lung cancer. Nat Rev Dis Primers. (2021) 7:3. doi: 10.1038/s41572-020-00235-0 33446664 PMC8177722

[2] Tariq S Kim SY Monteiro de Oliveira Novaes J Cheng H. Update 2021: management of small cell lung cancer. Lung. (2021) 199:579–87. doi: 10.1007/s00408-021-00486-y 34757446

[3] van Meerbeeck JP Fennell DA De Ruysscher DK. Small-cell lung cancer. Lancet. (2011) 378:1741–55. doi: 10.1016/S0140-6736(11)60165-7 21565397

[4] Farago AF Keane FK. Current standards for clinical management of small cell lung cancer. Transl Lung Cancer Res. (2018) 7:69–79. doi: 10.21037/tlcr.2018.01.16 29535913 PMC5835595

[5] Horn L Mansfield AS Szczęsna A Havel L Krzakowski M Hochmair MJ. First-line atezolizumab plus chemotherapy in extensive-stage small-cell lung cancer. N Engl J Med. (2018) 379:2220–9. doi: 10.1056/NEJMoa1809064 30280641

[6] Paz-Ares L Dvorkin M Chen Y Reinmuth N Hotta K Trukhin D. Durvalumab plus platinum-etoposide versus platinum-etoposide in first-line treatment of extensive-stage small-cell lung cancer (CASPIAN): a randomised, controlled, open-label, phase 3 trial. Lancet. (2019) 394:1929–39. doi: 10.1016/S0140-6736(19)32222-6 31590988

[7] Wang J Zhou C Yao W Wang Q Min X Chen G. Adebrelimab or placebo plus carboplatin and etoposide as first-line treatment for extensive-stage small-cell lung cancer (CAPSTONE-1): a multicentre, randomised, double-blind, placebo-controlled, phase 3 trial. Lancet Oncol. (2022) 23:739–47. doi: 10.1016/S1470-2045(22)00224-8 35576956

[8] Cheng Y Han L Wu L Chen J Sun H Wen G. Effect of first-line serplulimab vs placebo added to chemotherapy on survival in patients with extensive-stage small cell lung cancer: the ASTRUM-005 randomized clinical trial. JAMA. (2022) 328:1223–32. doi: 10.1001/jama.2022.16464 PMC9516323 36166026

[9] Lee WS Yang H Chon HJ Kim C. Combination of anti-angiogenic therapy and immune checkpoint blockade normalizes vascular-immune crosstalk to potentiate cancer immunity. Exp Mol Med. (2020) 52:1475–85. doi: 10.1038/s12276-020-00500-y PMC8080646 32913278

[10] Syed YY. Anlotinib: first global approval. Drugs. (2018) 78:1057–62. doi: 10.1007/s40265-018-0939-x 29943374

[11] Cheng Y Chen J Zhang W Xie C Hu Q Zhou N. Benmelstobart, anlotinib and chemotherapy in extensive-stage small-cell lung cancer: a randomized phase 3 trial. Nat Med. (2024) 30(10):2967–76. doi: 10.1038/s41591-024-03132-1 PMC11485241 38992123

[12] Husereau D Drummond M Augustovski F de Bekker-Grob E Briggs AH Carswell C. Consolidated health economic evaluation reporting standards 2022 (CHEERS 2022) statement: updated reporting guidance for health economic evaluations. Value Health. (2022) 25:3–9. doi: 10.1016/j.jval.2021.11.1351 35031096

[13] Liu L Wang L Chen L Ding Y Zhang Q Shu Y. Cost-effectiveness of sintilimab plus chemotherapy versus chemotherapy alone as first-line treatment of locally advanced or metastatic oesophageal squamous cell carcinoma. Front Immunol. (2023) 14:1092385. doi: 10.3389/fimmu.2023.1092385 36756110 PMC9899904

[14] Compiled by national bureau of statistics of China . Available online at: https://www.stats.gov.cn/sj/ndsj/2023/indexch.htm (Accessed August 1, 2024).

[15] Yue X Li Y Wu J Guo JJ. Current development and practice of pharmacoeconomic evaluation guidelines for universal health coverage in China. Value Health Reg Issues. (2021) 24:1–5. doi: 10.1016/j.vhri.2020.07.580 33349598

[16] Hoyle MW Henley W. Improved curve fits to summary survival data: application to economic evaluation of health technologies. BMC Med Res Methodol. (2011) 11:139. doi: 10.1186/1471-2288-11-139 21985358 PMC3198983

[17] Ishak KJ Kreif N Benedict A Muszbek N. Overview of parametric survival analysis for health-economic applications. Pharmacoeconomics. (2013) 31:663–75. doi: 10.1007/s40273-013-0064-3 23673905

[18] Williams C Lewsey JD Mackay DF Briggs AH. Estimation of survival probabilities for use in cost-effectiveness analyses: A comparison of a multi-state modeling survival analysis approach with partitioned survival and markov decision-analytic modeling. Med Decis Making. (2017) 37:427–39. doi: 10.1177/0272989X16670617 PMC5424853 27698003

[19] Yao ZH. The big data service platform for China’s health industry: Information Query of Drug Bid Winning. Available online at: https://data.yaozh.com/ (Accessed August 1, 2024).

[20] Zheng Z Chen H Cai H. Cost-effectiveness analysis of serplulimab combination therapy versus chemotherapy alone for patients with extensive-stage small cell lung cancer. Front Oncol. (2023) 13:1259574. doi: 10.3389/fonc.2023.1259574 38282674 PMC10812113

[21] Shen J Du Y Shao R Jiang R. First-line sintilimab plus chemotherapy in locally advanced or metastatic esophageal squamous cell carcinoma: A cost-effectiveness analysis from China. Front Pharmacol. (2022) 13:967182. doi: 10.3389/fphar.2022.967182 36569294 PMC9767976

[22] Peng Y Zeng X Peng L Liu Q Yi L Luo X. Sintilimab plus bevacizumab biosimilar versus sorafenib as first-line treatment for unresectable hepatocellular carcinoma: A cost-effectiveness analysis. Front Pharmacol. (2022) 13:778505. doi: 10.3389/fphar.2022.778505 35222020 PMC8864224

[23] Shao T Zhao M Tang W. Cost-effectiveness analysis of sintilimab vs. placebo in combination with chemotherapy as first-line therapy for local advanced or metastatic oesophageal squamous cell carcinoma. Front Oncol. (2022) 12:953671. doi: 10.3389/fonc.2022.953671 36561521 PMC9763586

[24] Wen F Zheng H Zhang P Liao W Zhou K Li Q. Atezolizumab and bevacizumab combination compared with sorafenib as the first-line systemic treatment for patients with unresectable hepatocellular carcinoma: A cost-effectiveness analysis in China and the United states. Liver Int. (2021) 41:1097–104. doi: 10.1111/liv.14795 33556230

[25] Huang Y You M Wu Q Chen R. Cost-effectiveness analysis of zolbetuximab plus mFOLFOX6 as the first-line treatment for CLDN18.2-positive, HER2-negative advanced gastric or Gastroesophageal Adenocarcinoma. Front Pharmacol. (2023) 14:1238009. doi: 10.3389/fphar.2023.1238009 37719841 PMC10500349

[26] Liu S Dou L Wang K Shi Z Wang R Zhu X. Cost-effectiveness analysis of nivolumab combination therapy in the first-line treatment for advanced esophageal squamous-cell carcinoma. Front Oncol. (2022) 12:899966. doi: 10.3389/fonc.2022.899966 35936686 PMC9353037

[27] Nafees B Lloyd AJ Dewilde S Rajan N Lorenzo M. Health state utilities in non-small cell lung cancer: An international study. Asia Pac J Clin Oncol. (2017) 13:e195–195e203. doi: 10.1111/ajco.12477 26990789

[28] Liu Q Tan C Yi L Wan X Peng L Li J. Cost-effectiveness analysis of pembrolizumab plus chemotherapy as first-line therapy for extensive-stage small-cell lung cancer. PloS One. (2021) 16:e0258605. doi: 10.1371/journal.pone.0258605 34780478 PMC8592441

[29] Zhang Q Wu P He X Ding Y Shu Y. Cost-effectiveness analysis of camrelizumab vs. Placebo added to chemotherapy as first-line therapy for advanced or metastatic esophageal squamous cell carcinoma in China. Front Oncol. (2021) 11:790373. doi: 10.3389/fonc.2021.790373 34926306 PMC8671697

[30] Hoyle M Green C Thompson-Coon J Liu Z Welch K Moxham T. Cost-effectiveness of temsirolimus for first line treatment of advanced renal cell carcinoma. Value Health. (2010) 13:61–8. doi: 10.1111/j.1524-4733.2009.00617.x 19804430

