# Supplementary material for: Cost-effectiveness analysis of benmelstobart, anlotinib, and chemotherapy in extensive-stage small-cell lung cancer
Source: Front Immunol. 2024 Nov 25;15:1477146. doi: 10.3389/fimmu.2024.1477146 (PMC11625741; doi:10.3389/fimmu.2024.1477146)
Supplement: Supplementary file 1 [file Table1.docx]

Supplementary Material

**Cost-effectiveness analysis of benmelstobart, anlotinib, and chemotherapy in extensive-stage small-cell lung cancer**

**1. Supplementary Table A. CHEERS 2022 Checklist.**

**2. Supplementary Table B. Comparison of survival models.**

**3. Supplementary Figure A. Results of the survival curve fit the BEN-AL-EC and PLB-EC group.**

**1.** **Supplementary Table A. CHEERS 2022 Checklist.**

| **Topic** | **No.** | **Item** | **Reported** |
| --- | --- | --- | --- |
| **Title** |  |  |  |
|  | 1 | Identify the study as an economic evaluation and specify the interventions being compared. | Yes |
| **Abstract** |  |  |  |
|  | 2 | Provide a structured summary that highlights context, key methods, results, and alternative analyses. | Yes |
| **Introduction** |  |  |  |
| **Background and objectives** | 3 | Give the context for the study, the study question, and its practical relevance for decision making in policy or practice. | Yes |
| **Methods** |  |  |  |
| **Health economic analysis plan** | 4 | Indicate whether a health economic analysis plan was developed and where available. | Yes |
| **Study population** | 5 | Describe characteristics of the study population (such as age range, demographics, socioeconomic, or clinical characteristics). | Yes |
| **Setting and location** | 6 | Provide relevant contextual information that may influence findings. | Yes |
| **Comparators** | 7 | Describe the interventions or strategies being compared and why chosen. | Yes |
| **Perspective** | 8 | State the perspective(s) adopted by the study and why chosen. | Yes |
| **Time horizon** | 9 | State the time horizon for the study and why appropriate. | Yes |
| **Discount rate** | 10 | Report the discount rate(s) and reason chosen. | Yes |
| **Selection of outcomes** | 11 | Describe what outcomes were used as the measure(s) of benefit(s) and harm(s). | Yes |
| **Measurement of outcomes** | 12 | Describe how outcomes used to capture benefit(s) and harm(s) were measured. | Yes |
| **Valuation of outcomes** | 13 | Describe the population and methods used to measure and value outcomes. | Yes |
| **Measurement and valuation of resources and costs** | 14 | Describe how costs were valued. | Yes |
| **Currency, price date, and conversion** | 15 | Report the dates of the estimated resource quantities and unit costs, plus the currency and year of conversion. | Yes |
| **Rationale and description of model** | 16 | If modelling is used, describe in detail and why used. Report if the model is publicly available and where it can be accessed. | Yes |
| **Analytics and assumptions** | 17 | Describe any methods for analysing or statistically transforming data, any extrapolation methods, and approaches for validating any model used. | Yes |
| **Characterising heterogeneity** | 18 | Describe any methods used for estimating how the results of the study vary for subgroups. | Yes |
| **Characterising distributional effects** | 19 | Describe how impacts are distributed across different individuals or adjustments made to reflect priority populations. | Yes |
| **Characterising uncertainty** | 20 | Describe methods to characterise any sources of uncertainty in the analysis. | Yes |
| **Approach to engagement with patients and others affected by the study** | 21 | Describe any approaches to engage patients or service recipients, the general public, communities, or stakeholders (such as clinicians or payers) in the design of the study. | Not applicable |
| **Results** |  |  |  |
| **Study parameters** | 22 | Report all analytic inputs (such as values, ranges, references) including uncertainty or distributional assumptions. | Yes |
| **Summary of main results** | 23 | Report the mean values for the main categories of costs and outcomes of interest and summarise them in the most appropriate overall measure. | Yes |
| **Effect of uncertainty** | 24 | Describe how uncertainty about analytic judgments, inputs, or projections affect findings. Report the effect of choice of discount rate and time horizon, if applicable. | Yes |
| **Effect of engagement with patients and others affected by the study** | 25 | Report on any difference patient/service recipient, general public, community, or stakeholder involvement made to the approach or findings of the study | Not applicable |
| **Discussion** |  |  |  |
| **Study findings, limitations, generalisability, and current knowledge** | 26 | Report key findings, limitations, ethical or equity considerations not captured, and how these could affect patients, policy, or practice. | Yes |
| **Other relevant information** |  |  |  |
| **Source of funding** | 27 | Describe how the study was funded and any role of the funder in the identification, design, conduct, and reporting of the analysis | Yes |
| **Conflicts of interest** | 28 | Report authors conflicts of interest according to journal or International Committee of Medical Journal Editors requirements. | Yes |

**2.** **Supplementary Table B. Comparison of survival models.**

|  | AIC | | BIC | |
| --- | --- | --- | --- | --- |
|  | BEN-AL-EC group | PLB-EC group | BEN-AL-EC group | PLB-EC group |
| OS |  |  |  |  |
| Exponential | 800.993 | 1007.651 | 804.498 | 1011.161 |
| Gamma | 781.509 | 960.108 | 788.520 | 967.126 |
| Gen.F | 777.681 | 955.915 | 791.702 | 969.952 |
| Gen.gamma | 779.743 | 962.081 | 790.259 | 972.609 |
| Gompertz | 795.290 | 979.554 | 802.301 | 986.572 |
| Weibull | 784.273 | 961.860 | 791.284 | 968.879 |
| Log-logistic | 773.368 | 952.247 | 786.379 | 964.266 |
| Log-normal | 777.746 | 969.748 | 784.757 | 976.767 |
| PFS |  |  |  |  |
| Exponential | 952.649 | 952.649 | 956.154 | 956.154 |
| Gamma | 900.111 | 900.111 | 907.122 | 907.122 |
| Gen.F | 885.507 | 885.507 | 899.529 | 899.529 |
| Gen.gamma | 875.570 | 875.570 | 886.086 | 886.086 |
| Gompertz | 945.798 | 945.798 | 952.809 | 952.809 |
| Weibull | 914.828 | 914.828 | 921.839 | 921.839 |
| Log-logistic | 872.964 | 872.637 | 879.974 | 879.974 |
| Log-normal | 880.404 | 880.404 | 887.415 | 887.415 |

AIC, Akaike information criterion; BEN-AL-EC, benmelstobart combined with anlotinib plus etoposide/cisplatin; BIC, Bayesian information criterion; OS, overall survival; PFS, progression-free survival; PLB-EC, placebo combined with etoposide/cisplatin.

**3.** **Supplementary** **Figure A. Results of the survival curve fit the BEN-AL-EC and PLB-EC group.**


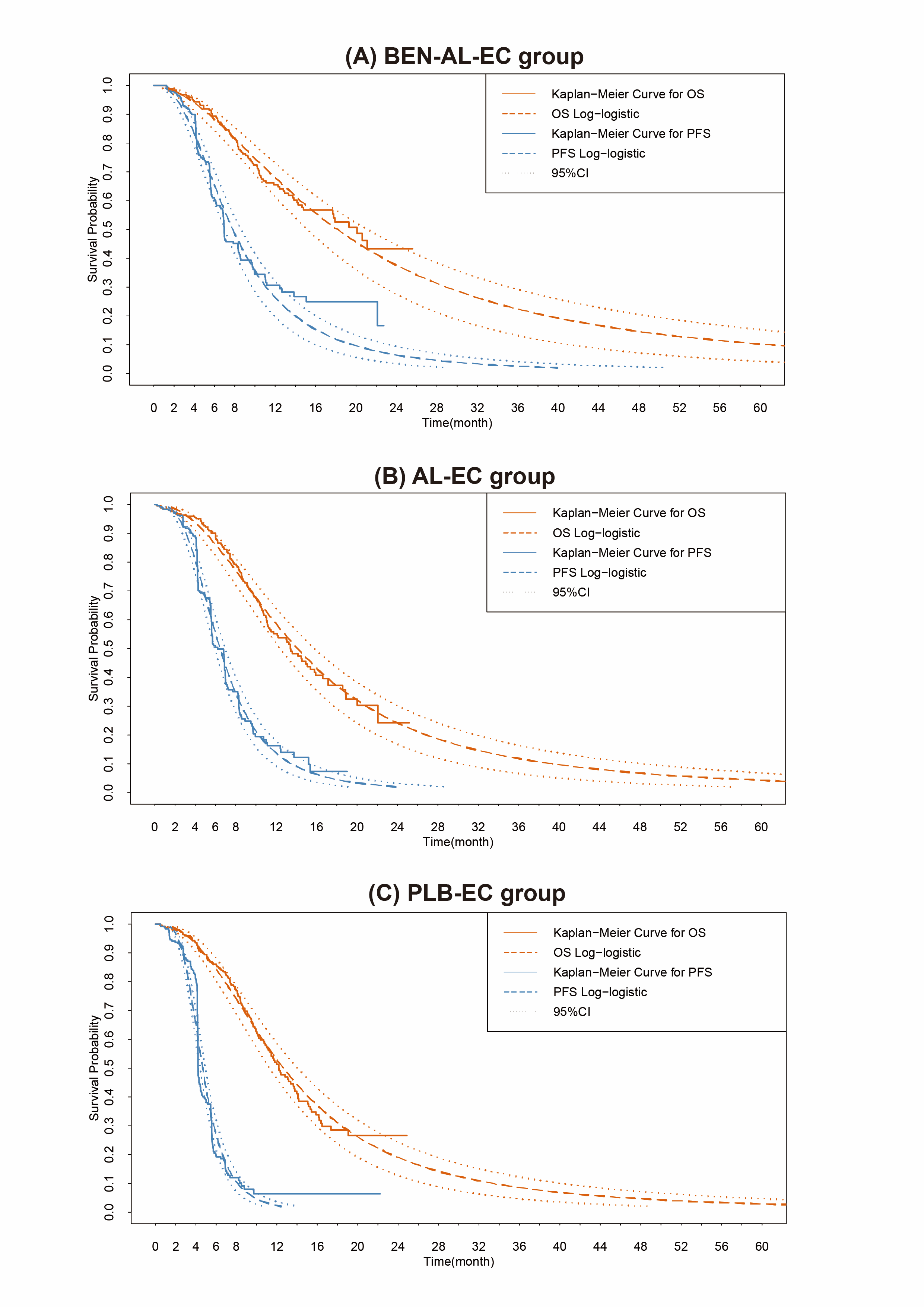


95%CI: 95% confidence interval; AL-EC, anlotinib plus etoposide/cisplatin; BEN-AL-EC, benmelstobart combined with anlotinib plus etoposide/cisplatin; OS, overall survival; PFS, progression-free survival; PLB-EC, placebo combined with etoposide/cisplatin.
